# Horse Oil Mitigates Oxidative Damage to Human HaCaT Keratinocytes Caused by Ultraviolet B Irradiation

**DOI:** 10.3390/ijms20061490

**Published:** 2019-03-25

**Authors:** Mei Jing Piao, Kyoung Ah Kang, Ao Xuan Zhen, Hee Kyoung Kang, Young Sang Koh, Bong Seok Kim, Jin Won Hyun

**Affiliations:** 1 School of Medicine, Jeju National University, Jeju 63243, Korea; meijing0219@hotmail.com (M.J.P.); legna48@hanmail.net (K.A.K.); zhenaoxuan705@gmail.com (A.X.Z.); pharmkhk@jejunu.ac.kr (H.K.K.); yskoh7@jejunu.ac.kr (Y.S.K.); 2 Bio Convergence Center, Jeju Technopark, Jeju 63243, Korea; kbs6953@jejutp.or.kr

**Keywords:** horse oil, ultraviolet B radiation, oxidative stress, apoptosis

## Abstract

Horse oil products have been used in skin care for a long time in traditional medicine, but the biological effects of horse oil on the skin remain unclear. This study was conducted to evaluate the protective effect of horse oil on ultraviolet B (UVB)-induced oxidative stress in human HaCaT keratinocytes. Horse oil significantly reduced UVB-induced intracellular reactive oxygen species and intracellular oxidative damage to lipids, proteins, and DNA. Horse oil absorbed light in the UVB range of the electromagnetic spectrum and suppressed the generation of cyclobutane pyrimidine dimers, a photoproduct of UVB irradiation. Western blotting showed that horse oil increased the UVB-induced Bcl-2/Bax ratio, inhibited mitochondria-mediated apoptosis and matrix metalloproteinase expression, and altered mitogen-activated protein kinase signaling-related proteins. These effects were conferred by increased phosphorylation of extracellular signal-regulated kinase 1/2 and decreased phosphorylation of p38 and c-Jun N-terminal kinase 1/2. Additionally, horse oil reduced UVB-induced binding of activator protein 1 to the matrix metalloproteinase-1 promoter site. These results indicate that horse oil protects human HaCaT keratinocytes from UVB-induced oxidative stress by absorbing UVB radiation and removing reactive oxygen species, thereby protecting cells from structural damage and preventing cell death and aging. In conclusion, horse oil is a potential skin protectant against skin damage involving oxidative stress.

## 1. Introduction

In many Asian countries including Korea, horse oil has long been used as a folk medicine. The efficacy of horse oil in hair care, beauty, and scar healing is described in classical medicine books such as “Mingyi Bielu”, “Compendium of Materia Medica”, and “Huang di’s Canon of Medicine”; however, few studies have examined these effects using modern techniques. Analysis of the ester fraction of horse oil revealed that C18 unsaturated acids, comprising 57% of total fatty acids, include oleic acid (60%), hepta-decadiene-carboxylic acid (linoleic acid isomer; 10%), and hepta-decatriene-carboxylic (linolenic) acid (30%) [1]. Recent studies have shown that oleic acid has anti-oxidative [2,3,4] and anti-inflammatory effects [5] as well as inhibits matrix metalloproteinases (MMPs) [6]. 

Both ultraviolet (UV) A and UVB reach the surface of the earth and penetrate the skin, inducing the production of a large amount of reactive oxygen species (ROS), thereby disrupting the redox balance in the organism to induce oxidative stress [7,8]. Many studies have demonstrated that UVB damages the skin by inducing oxidative stress, leading to a range of skin conditions including erythema [9], burns [10], inflammation [11], photo-aging [12], photo-carcinogenesis [13], and ultimately skin cancer [14]. Moreover, UVB is directly absorbed by DNA and generates pyrimidine adducts, such as cyclobutane pyrimidine dimer (CPD) and pyrimidine-pyrimidone (6-4) photoproducts [15], which are easily mutable substances [16]. MMPs are a family of matrix-degrading enzymes with important roles in various disruptive events, such as inflammation, cancer invasion and metastasis, and skin senescence [17,18]. Various studies have demonstrated that UVB-induced expression of MMPs (MMP-1, MMP-2, MMP-9) leads to collagen degradation [19,20,21]. UVB irradiation can mediate apoptosis via oxidative stress-dependent phosphorylation of upstream mitogen-activated protein kinases (MAPKs) in human HaCaT keratinocytes. Moreover, UV-generated ROS have been shown to stimulate the MAPK signaling pathway and transcription factor activator protein 1 (AP-1), leading to upregulation of MMP expression [22].

Here, we evaluated the protective effect of horse oil against UVB-induced photo-oxidative damage and photo-aging in human HaCaT keratinocytes.

## 2. Results

### 2.1. Analysis of Horse Oil Composition

According to the composition analysis report of the Korea Food Research Institute, the occupancy ratios of saturated fat in horse oil fat are 30.3% for HO-I and 42.1% for HO-II. The main ingredient is palmitic acid, accounting for 23.8% and 29.4% in HO-I and HO-II, respectively. The ratios of unsaturated fat in HO-I and HO-II are 69.3% and 57.1%, respectively, with oleic acid and linoleic acid as the major unsaturated fatty acids. The ratios of oleic acid and linoleic acid are 37.7% and 19.7% in HO-I and 32.5% and 15.0% in HO-II, respectively (Table 1).

### 2.2. Effect of Horse Oil on UVB-Induced ROS Generation

**Figure 1:**
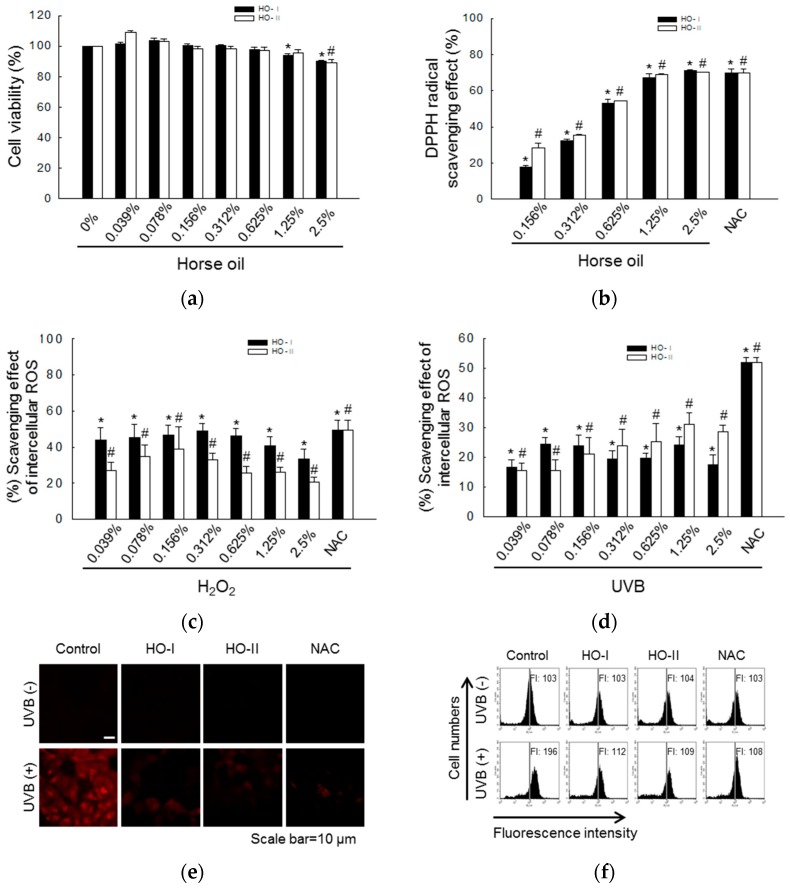
Horse oil can scavenge stress-induced ROS. (**a**) Horse oil (HO-I or HO-II) was added at the indicated final concentrations. After 24 h, cell viability was measured by MTT assay. (**b**) DPPH radical levels were measured spectrophotometrically at 520 nm. *, # *p* < 0.05 vs. non-treated control cells. Intracellular ROS levels generated by (**c**) H_2_O_2_ or (**d**) UVB radiation were detected by spectrofluorometry after H_2_DCFDA staining. NAC served as a positive control. * *p* < 0.05 vs. H_2_O_2_ alone, and # *p* < 0.05 vs. UVB alone. Cells were treated with 0.312% horse oil for 1 h, and then with UVB radiation at 30 mJ/cm^2^. Next, the cells were incubated for 24 h, and intracellular ROS were detected by (**e**) confocal microscopy (Scale bar = 10 μm) and (**f**) flow cytometry after H_2_DCFDA staining.

Horse oil showed no toxicity towards human HaCaT keratinocytes at a concentration equal to or lower than 0.625%; however, HO-I was cytotoxic at concentrations above 1.25% and HO-II was cytotoxic at concentrations above 2.5% (Figure 1a). In cell-free systems, horse oil scavenges DPPH free radicals in a concentration-dependent manner. HO-I and HO-II showed the best free radical scavenging effects of 71% and 70%, respectively, which were equivalent to 70% of the positive control *N*-acetylcysteine (NAC), at the highest concentration of 2.5% (Figure 1b). In the cell system, horse oil also effectively scavenged H_2_O_2_ or UVB-induced ROS. HO-I and HO-II at 0.312% exhibited the best scavenging effect in H_2_O_2_ test groups, scavenging 49% and 33% of ROS, respectively. For UVB test groups, the strongest effect was observed at a concentration of 1.25%, reducing ROS by 24% and 31%, respectively. These effects were close to the 50% or 52% scavenging effects of NAC (Figure 1c,d). Considering the cytotoxicity and scavenging effects, we selected 0.312% as the optimal concentration of horse oil for further experiments. Confocal microscopy revealed that the red fluorescence intensity of dichlorodihydrofluorescein produced by the reaction of H_2_DCFDA and ROS was significantly reduced in 0.312% horse oil-pretreated cells compared to UVB-irradiated cells (Figure 1e). Flow-cytometric analysis of UVB-irradiated ROS levels revealed that the fluorescence intensity in the UVB-exposed group was 196, whereas those in the cells pretreated with HO-I and HO-II were 112 and 109, respectively (Figure 1f).

### 2.3. Effect of Horse Oil on UVB-Induced Macromolecular Damage

**Figure 2:**
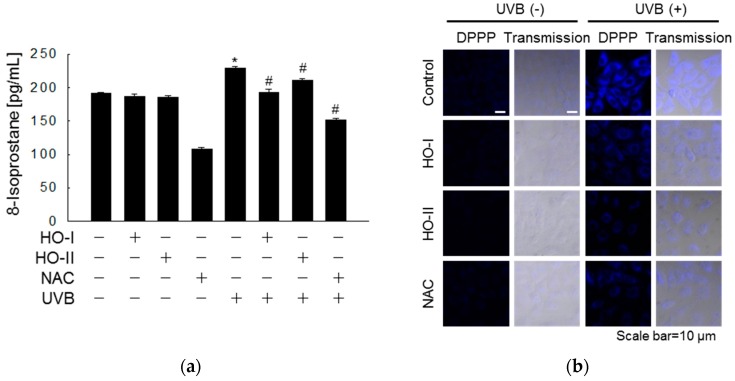

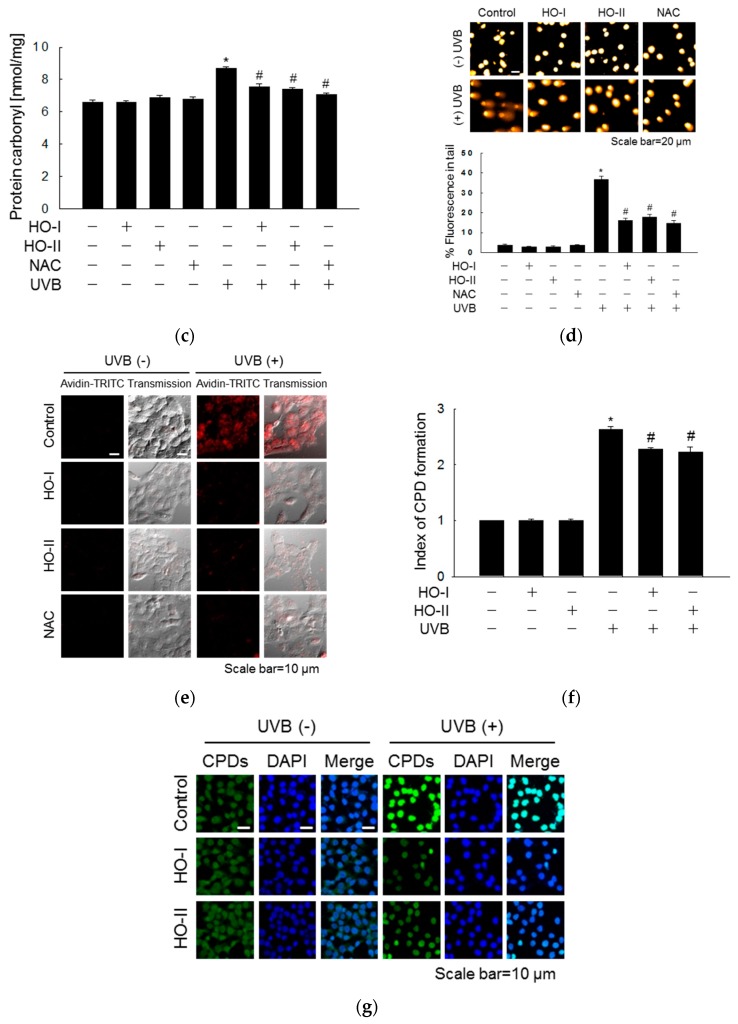
Horse oil mitigates UVB-induced oxidative damage to cellular macromolecules. Cells were treated with horse oil or NAC for 1 h and then exposed to UVB radiation. After incubation, lipid peroxidation was assayed by measuring (**a**) the levels of 8-isoprostane secreted into the culture medium or (**b**) DPPP-stained cells were detected by confocal microscopy and quantified (Scale bar=10 μm). (**c**) Protein oxidation was assayed by measuring protein carbonylation. (**d**) DNA damage was assessed by the comet assay. Representative images and the percentage of total cellular DNA in comet tails are shown (Scale bar=10 μm). * *p* < 0.05 vs. control, and # *p* < 0.05 vs. UVB-irradiated cells. (**e**) 8-OxoG detected by the binding of avidin-TRITC was visualized by confocal microscopy (Scale bar=10 μm). DNA was extracted and analyzed by (**f**) ELISA and (**g**) immunocytochemistry using an antibody against CPDs. DAPI was used to stain the nuclei (Scale bar = 10 μm). * *p* < 0.05 vs. control, and # *p* < 0.05 vs. UVB-irradiated cells.

We next evaluated UVB-mediated oxidative damage of cellular structures and the protective properties of horse oil by assessing the extent of lipid peroxidation, protein carbonyl formation, and DNA strand breaks. Lipid peroxidation was evaluated by detecting total 8-isoprostane in conditioned cell culture media. Cells exposed to UVB showed increased 8-isoprostane levels (229 pg/mL) compared to the control group (191 pg/mL). However, pretreatment with horse oil (193 and 210 pg/mL) attenuated the UVB-induced elevation in 8-isoprostane levels (Figure 2a). The fluorescence intensity of DPPP oxide was stronger in UVB-irradiated cells than in control cells. However, pretreatment of cells with horse oil considerably reduced lipid peroxidation in UVB-irradiated cells, as did pretreatment with NAC (Figure 2b). Protein carbonylation is a form of oxidative protein damage promoted by UVB-generated ROS. The protein carbonyl content of cells exposed to UVB (8.7 nmol/mg) was noticeably higher than that in control cells; however, HO-I and HO-II pretreatment significantly reduced the protein carbonyl content to 7.6 and 7.4 nmol/mg, respectively. The effects of the horse oils were similar to that of NAC, which reduced the protein carbonyl content to 7.1 nmol/mg (Figure 2c). Finally, an alkaline comet assay was performed to visualize DNA damage in cells resulting from UVB irradiation. Exposure of keratinocytes to UVB irradiation increased the number of DNA strand breaks, which increased the fluorescence intensity in the tails of comet-like structures formed during the comet assay. Obvious comet tail formation was observed in UVB-treated cells compared to in control cells, but horse oil and NAC pretreatment both attenuated this phenomenon. Quantification in image analysis revealed that the portion of total cellular DNA in tails in cells exposed to UVB was 37%, while those in the HO-I-, HO-II-, and NAC-pretreated UVB-irradiated cells were 16%, 18%, and 15%, respectively (Figure 2d). The level of 8-oxoG, a hallmark of oxidative stress-induced DNA base damage, was detected in an avidin-TRITC assay and verified by confocal microscopy. The fluorescence intensity in UVB-irradiated cells treated with horse oil was significantly weaker than that in cells irradiated with UVB alone (Figure 2e). Because UV light induced the production of CPDs, which represent 70–80% of total UV-induced photoproducts, the effect of horse oil on UVB-induced CPD production was detected. The CPD level in UVB-exposed cells was 2.63-fold higher than that in control cells, as indicated by enzyme-linked immunosorbent assay (ELISA); however, horse oil pretreatment significantly inhibited the UVB-induced CPD level by up to 2.27- and 2.23-fold, respectively (Figure 2f). Immunocytochemical analysis confirmed this finding (Figure 2g).

### 2.4. Effect of Horse Oil on UVB-Induced Apoptosis and MAPK Signaling

**Figure 3:**
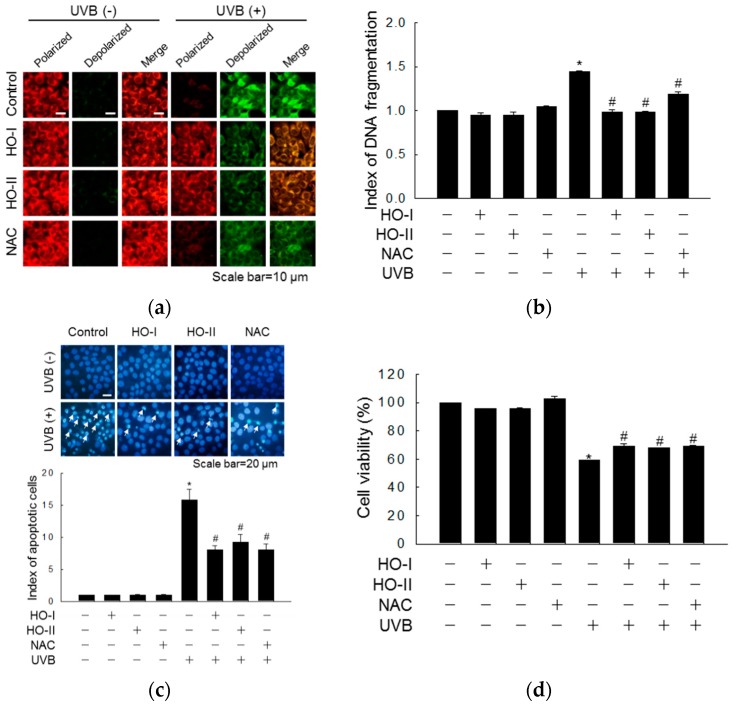

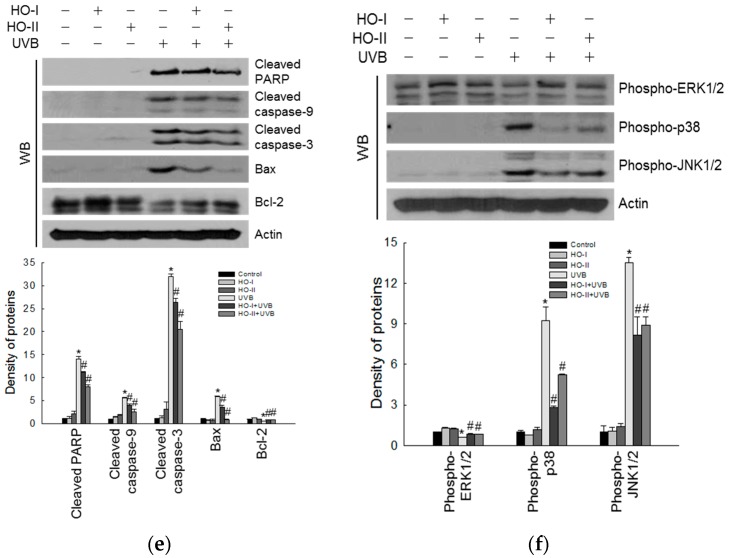
Horse oil attenuates UVB-induced apoptosis. HaCaT keratinocytes were treated with horse oil or NAC and exposed to UVB radiation 1 h later. Cells were then incubated for 24 h. Δψm was analyzed by (**a**) confocal microscopy after staining the cells with JC-1 (Scale bar = 10 μm). (**b**) DNA fragmentation was quantified by ELISA. (**c**) Apoptotic bodies (arrows) were observed in cells stained with Hoechst 33342 dye and quantified by fluorescence microscopy (Scale bar = 10 μm). (**d**) Cell viability following UVB radiation was determined by MTT assay. (**e**,**f**) Western blotting with antibodies specific for (**e**) PARP, caspase-9, caspase-3, Bax, Bcl-2, actin, (**f**) phospho-ERK1/2, phospho-p38, and phospho-JNK1/2, and actin, and the results were quantified (*n* = 3). * *p* < 0.05 vs. control, and # *p* < 0.05 vs. UVB-irradiated cells.

As shown in the fluorescence micrographs in Figure 3a, control cells showed bright red fluorescence, indicating mitochondrial membrane polarization. However, UVB exposure reduced the intensity of red fluorescence and increased the intensity of green fluorescence, revealing mitochondrial membrane depolarization. In horse oil- and NAC-pretreated cells, the reduction in red fluorescence was inhibited (Figure 3a). Furthermore, while the amount of cytoplasmic histone-associated DNA fragments was 1.44-fold higher in UVB-irradiated than in control cells, the level of DNA fragmentation was significantly suppressed by 0.99-, 0.99-, and 1.19-fold in horse oil- and NAC-treated cells (Figure 3b). Next, to investigate the cytoprotective properties of horse oil against UVB-induced apoptosis, the cell nuclei were stained with Hoechst 33342 and observed by fluorescence microscopy. We previously demonstrated the UVB-induced apoptosis of keratinocytes in the presence of apoptotic bodies [23]. Numerous apoptotic bodies were detected in the UVB irradiation group, whereas apoptotic bodies in horse oil- or NAC-pretreated cells were significantly reduced (Figure 3c). Next, we investigated whether horse oil impacted the viability of UVB-irradiated keratinocytes. An MTT assay showed that the cell viabilities of horse oil- and NAC-pretreated cells were restored from 59.1% to 69.5%, 67.9%, and 69.2%, after UVB irradiation (Figure 3d). Caspase-9 is cleaved upon mitochondrial membrane disruption [24]. This active (cleaved) form of the enzyme and of its target, caspase-3, were evaluated by Western blotting. Horse oil suppressed the UVB-induced cleavage of caspase-9 (39 and 37 kDa) and cleaved caspase-3 (19 and 17 kDa), as further demonstrated by activation of PARP (89 kDa) (Figure 3e). In keratinocytes, phosphorylation of JNK1/2 and p38 MAPK is activated UVB-induced apoptosis [25], whereas extracellular signal-regulated kinases 1 and 2 (ERK1/2) are related to the cell survival pathway and their inhibition promotes UVB-induced apoptosis [26]. As shown in Figure 3f, horse oil significantly inhibited the phosphorylation of p38 and JNK after UVB irradiation. However, the UVB-dependent decrease in phospho-ERK was reversed by horse oil. These results indicate that horse oil protects against cell apoptosis controlled by MAPK signaling by inhibiting the mitochondrial caspase-dependent pathway.

### 2.5. Effect of Horse Oil on UVB Absorption

**Figure 4:**
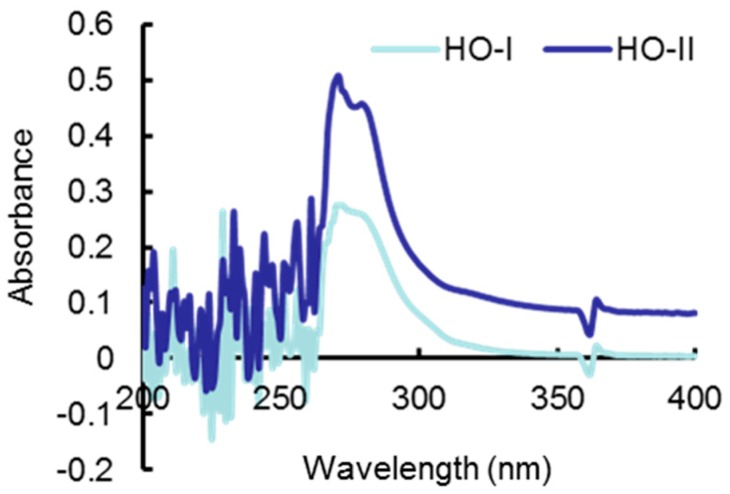
Horse oil absorbs UVB rays. The UVB absorption spectrum of horse oil was determined by UV scanning at 200–400 nm. The maximum absorbance of horse oil appeared at 271 nm, and the absorbance values of HO-I or HO-II were 0.2756 and 0.5067, respectively.

Horse oil showed a high absorption ability in the UV range (200–400 nm), reaching a peak at 271 nm, with absorption values of 0.28 and 0.51 for HO-I and HO-II, respectively (Figure 4). The absorbance of this value indicates that the horse oil has UVB light absorption properties, but does not completely prevent UVB ray from reaching the cells.

### 2.6. Effect of Horse Oil on UVB-Induced MMP Expression and Activation

**Figure 5:**
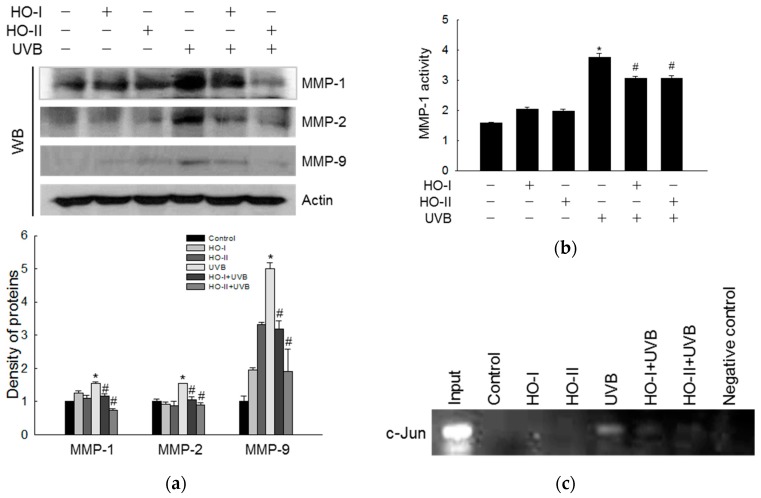
Horse oil reduces UVB-induced MMP expression and activation. (**a**) Western blot analysis of MMP-1, MMP-2, and MMP-9 protein expression and quantitative data are shown (*n* = 3). (**b**) Active MMP-1 was quantified in the culture supernatants. Cells were incubated in serum-free medium to eliminate interference from MMP-1 in the serum. * *p* < 0.05 vs. control, and # *p* < 0.05 vs. UVB-irradiated cells. (**c**) AP-1 binding to the MMP-1 promoter was assessed by ChIP assay.

It has been reported that the activities of MMP-1, MMP-2, MMP-3, and MMP-9 are evidently increased in UV-induced photo-aging skin [27,28]. Horse oil inhibited the induction of MMP-1, MMP-2, and MMP-9 protein expression by UVB irradiation (Figure 5a). Furthermore, horse oil significantly inhibited the activity of MMP-1 induced by UVB radiation from 3.75 ng/mL to 3.06 ng/mL and 3.06 ng/mL, respectively (Figure 5b). These results suggested that horse oil markedly suppressed the activation of MMP enzymes. AP-1, a heterodimeric protein consisting of proteins belonging to the Fos and Jun families, is a transcription factor capable of binding to 5′-TGAGTCA-3′ DNA elements [29]. The AP-1 site plays an important role in the transcriptional activation of MMP promoters [30]. A chromatin immunoprecipitation (ChIP) assay revealed that horse oil attenuated UVB-induced binding of AP-1 to the MMP-1 promoter (Figure 5c).

## 3. Discussion

To provide a scientific basis for the skin-protective effect of horse oil, we focused on the protective mechanism of horse oil on UVB-induced oxidative stress in human HaCaT keratinocytes. The skin utilizes an effective antioxidant defense system that responds to UV-induced oxidative stress, including that caused by UVB exposure. However, excessive and prolonged exposure to UV radiation may overwhelm the skin’s antioxidant system, leading to oxidative damage resulting in skin disorders such as sunburn, photosensitivity, and precancerous growth. As described above, UVB radiation can reach the epidermal basal cell layer of the skin; thus, its effect is most pronounced in epidermal cells, including keratinocytes. 

Fatty acid analysis of the horse oils indicated that, although the two horse oils differ in fatty acid contents, they contain the same type of fatty acids. They are rich in natural nutrients, such as oleic acid, linoleic acid, palmitic acid, and other highly unsaturated fatty acids. The different contents may be mainly related to the different product formulations (Table 1). Extraction methods differ depending on the manufacturer’s equipment, skill level, and other factors. Smear-type horse oil and dissolved horse oil have different effects, such as their degree of skin absorption. In the current study, we tested two types of horse oil at different dosages to study the protective effect of horse oils on UVB-induced cell damage. 

The UV absorption characteristics of horse oil in Figure 4 indicate that horse oil has the ability to block UVB light from reaching the cell. However, its absorption capacity is not very strong at 0.312% concentration of horse oil, so the UV absorption properties of horse oil are not a major protective effect. We suggest that its protective effect is mainly caused by the antioxidant action of horse oil and the resulting changes in cell signaling processes. We demonstrated that horse oil scavenges DPPH free radicals in cell-free systems and scavenges H_2_O_2_ and UVB-induced intracellular ROS (Figure 1). 

If excessive ROS are not rapidly removed, they may attack biological macromolecules, such as lipids, proteins, and DNA, resulting in extensive cellular oxidative damage that can disrupt cell function and contribute to cell death. However, some substances show cytoprotective effects against oxidative stress-induced cell damage induced by UVB exposure. The current study showed that horse oil significantly prevents UVB-induced peroxidation damage to lipid membranes and reduces the level of UVB-enhanced protein carbonylation. Additionally, horse oil significantly reduced oxidative DNA damage caused by UVB rays. Finally, horse oil showed significant protective effects against the major UVB-induced photoproduct, CPDs. 

Notably, after exposure to UVB, apoptotic body formation and cell death signaling were stimulated in HaCaT keratinocytes. Horse oil pretreatment effectively inhibited apoptosis after UVB irradiation, likely by downregulating PARP, caspase-3, caspase-9, and Bax and upregulating Bcl-2. Further, MAPK signaling plays an important role in controlling cell proliferation, cell motility, MMP gene expression, and cell survival and death. The three major MAPK subfamilies in mammalian cells are JNK, ERK, and p38 [31]. Oxidative stress caused by ROS accumulation can initiate MAPK signaling through the phosphorylation of MAPK members [32]. This study revealed that UVB-induced JNK and p38 activation was reduced in horse oil-pretreated cells, while UVB-suppressed ERK activity was restored. 

MMP-1 is a member of the collagenase subfamily of MMPs. Previous studies showed that UVB-induced ROS cause skin aging by activating MMP-1 [17]. Upon activation, MMP-1 initiates collagen breakdown by cleaving type I and type III collagens, which are then cleaved by MMP-2 and MMP-9 [33]. The gelatinolytic activities of MMP-2 and MMP-9 are central players in UV-irradiated skin damage and the formation of wrinkles [34]. A previous study suggested that antioxidants suppress UVB-induced MMP-9 activity in human HaCaT keratinocytes [35]. The MMP-1 promoter contains a binding site for AP-1, and UVB-induced AP-1 activation amplifies MMP-1 expression [36]. Accordingly, the present data show that horse oil can inhibit UVB-induced MMPs, such as MMP-1, MMP-2, and MMP-9, and suppresses UVB-induced AP-1 transcriptional activity in the MMP-1 promoter region.

In our system, oleic acid, which is the most abundant component of horse oil, showed a DPPH free radical-scavenging capacity of less than 10% in a cell-free system and showed no ability to scavenge intracellular ROS induced by H_2_O_2_ and UVB. Oleic acid also did not show absorption capacity at the UVB wavelength (data not shown). The second most unsaturated fatty acid in horse oil, linoleic acid, inhibits peroxidation, DNA damage, and apoptosis [37,38]. Our future studies will focus on identifying the fatty acids responsible for the protective effects of horse oil against oxidative stress-induced cell damage. The effects of single active ingredients of horse oil on cells have been reported previously. However, horse oil is a mixture of unsaturated and saturated fatty acids, and studies on their effects on cells are rare. The protective effect of horse oil on UV-induced skin may be mediated by various active ingredients. Further studies are needed to determine the molecular and pharmacodynamic mechanisms of horse oil in vitro and in vivo. 

## 4. Materials and Methods

### 4.1. Preparation of Horse Oil

Horse oils were provided by Beihai Company (Jeju, Korea) and Daebong LS Company (Jeju). Horse oil (HO-I) provided by Beihai Company is a liquid product made from 100% pure horse oil extracted by vacuum distillation of horse fat. Horse oil (HO-II) provided by Daebong LS Company is a cream product made from 100% pure horse oil extracted from the water vapor of horse fat. The horse oil stock solution was diluted with horse oil stock solution:distilled water:Tween-80 at a ratio of 1:1:0.02, and the milky horse oil was obtained by ultrasonic treatment and then treated to the cells.

### 4.2. Fatty Acid Analysis of Horse Oil

The fatty acid content of horse oil was analyzed according to the AOAC official method 963.22 of Korea Food Research Institute (Jeollabuk-do, Korea).

### 4.3. Cell Culture and UVB Radiation

Human HaCaT keratinocytes were provided by CLS Cell Lines Service GmbH (Eppelheim, Germany). Cells were cultured in Dulbecco’s modified Eagle’s medium containing 10% heat-inactivated fetal bovine serum (Life Technologies, Carlsbad, CA, USA) and antibiotic-antimycotic (Life Technologies) at 37 °C in 5% CO_2_. The cells were irradiated with UVB at a dose of 30 mJ/cm^2^. After the cells were irradiated with UVB light, the original medium was replaced with new medium, and the experiment was carried out after incubation. The UVB source was a CL-1000M UV Crosslinker (UVP, Upland, CA, USA), which was used to deliver an energy spectrum corresponding to UVB wavelengths of 280–320 nm.

### 4.4. Cell Viability Assay

Cells were seeded into 24-well plates at 0.315 × 10^5^ cells per cm^2^ (0.6 × 10^5^ cells/well). After 16 h of incubation, the cells were treated with 0.039%, 0.078%, 0.156%, 0.312%, 0.625%, 1.25%, or 2.5% horse oil (final concentrations) or pretreated with horse oil (0.312%) and the positive control *N*-acetylcysteine (1 mM, NAC, Sigma-Aldrich, St. Louis, MO, USA). One hour later, the cells were UVB-irradiated. After one day of incubation at 37 °C, 50 μL of thiazolyl blue tetrazolium bromide (MTT, Amresco LLC, Solon, OH, USA) stock solution (2 mg/mL) was added to each well. After 4 h, the formazan crystals in each well were dissolved in 350 μL of dimethyl sulfoxide (Amresco LLC) and the absorbance at 540 nm was read on a VersaMax ELISA Microplate Reader (Molecular Devices, Sunnyvale, CA, USA) [39].

### 4.5. Detection of 2,2-Diphenyl-1-picrylhydrazyl (DPPH) Radicals

Horse oil (0.156%, 0.312%, 0.625%, 1.25%, and 2.5%, final concentrations) and positive control NAC (2 mM) were added to 96-well plates containing 0.15 mM DPPH (Sigma-Aldrich) in ethanol. The reaction mixture was shaken vigorously; after 3 h, the amount of DPPH remaining was detected at 520 nm. The DPPH radical-scavenging activity (%) was calculated as: [(optical density of DPPH radical treatment) – (optical density of horse oil or NAC with DPPH radical treatment)] × 100/(optical density of DPPH radical treatment).

### 4.6. Detection of Intracellular ROS

Cells were seeded into 96-well plates at 0.47 × 10^5^ cells per cm^2^ (0.15 × 10^5^ cells/well) (H_2_O_2_) or 0.52 × 10^5^ cells per cm^2^ (0.1 × 10^5^ cells/well) (UVB). After 16 h, 0.039%, 0.078%, 0.156%, 0.312%, 0.625%, 1.25%, or 2.5% horse oil (final concentrations) and 1 mM NAC were added, and the plates were incubated at 37 °C for 30 min or 1 h. Next, the cells were exposed to H_2_O_2_ (500 μM) or UVB light, and then further incubated at 37 °C for 30 min or 24 h. A 2′,7′-dichlorodihydrofluorescein diacetate (H_2_DCFDA; Molecular Probes, Eugene, OR, USA) solution (25 µM) was added, and the cells were incubated for 10 min. DCF fluorescence was measured with a PerkinElmer LS-5B spectrofluorometer (Waltham, MA, USA). For image analysis, cells were seeded onto a 4-well glass slide at 0.35 × 10^5^ cells per cm^2^ (0.6 × 10^5^ cells/well) and cultured for 16 h. The cells were treated with horse oil (0.312%) and NAC (1 mM) for 1 h, exposed to UVB, and incubated for another 24 h at 37 °C. H_2_DCFDA (100 µM) was added to each well, and the stained cells were assessed under a FV1200 laser scanning microscope (Carl Zeiss, Jena, Germany). For flow cytometry analysis, cells were seeded into a 6-well plate at 0.21 × 10^5^ cells per cm^2^ (2.0 × 10^5^ cells/well). The cells were treated with horse oil and NAC under the above conditions. After incubation with H_2_DCFDA at 37 °C for 30 min, the cells were analyzed with a BD FACSCalibur flow cytometer (BD Biosciences, San Jose, CA, USA). 

### 4.7. Lipid Peroxidation Assay

The level of 8-isoprostane, a marker of lipid peroxidation, was measured using a commercial enzyme immunoassay (Cayman Chemical, Ann Arbor, MI, USA) according to the manufacturer’s protocol [40]. Additionally, lipid peroxidation was assessed by using fluorescent probe diphenyl-1-pyrenylphosphine (DPPP; Molecular Probes). Cells were incubated with 5 µM DPPP for 15 min in the dark, washed with PBS, and mounted on a microscope slide in mounting medium (DAKO, Carpinteria, CA, USA). DPPP fluorescence was analyzed under FV1200 laser-scanning microscope at an excitation wavelength of 351 nm and emission wavelength of 380 nm.

### 4.8. Protein Carbonyl Formation

The formation of protein carbonyl, a marker of protein oxidation, was assessed by using an Oxiselect^TM^ protein carbonyl ELISA kit (Cell Biolabs, San Diego, CA, USA) according to the manufacturer’s protocol. 

### 4.9. Single-Cell Gel Electrophoresis (Comet Assay)

DNA oxidation was assessed in an alkaline comet assay. A cell suspension was mixed with 0.7% low-melting agarose at 39 °C and spread onto a fully frosted microscope slide pre-coated with 1% normal melted agarose. After curation, the slides were covered with 0.7% low melting agarose and immersed in lysis buffer (2.5 M NaCl, 100 mM Na_2_EDTA, 10 mM Tris-pH10, 1% *N*-lauroylsarcosinate, and 1% Triton X-100) at 4 °C for 1.5 h. Electrophoresis was carried out for 20 min at 300 mA, 25 V, and the gel was stained with ethidium bromide. The percentage of DNA in the comet tail for each cell was recorded as described previously [23]. 

### 4.10. Detection of 8-Oxoguanine (8-oxoG)

8-OxoG, a marker of oxidative DNA damage, was evaluated in a fluorescent binding assay [41]. Cells were seeded onto a 4-well chamber slide at 0.35 × 10^5^ cells per cm^2^ (0.6 × 10^5^ cells/well). After 16 h of incubation, the cells were treated with horse oil (0.312%) and NAC (1 mM) for 1 h and then exposed to UVB and incubated at 37 °C for 24 h. The cells were fixed and permeabilized with ice-cold methanol for 15 min, and the 8-oxoG level was determined by measuring fluorescence under an FV1200 laser-scanning microscope after a reaction with avidin-TRITC conjugate (Sigma-Aldrich).

### 4.11. CPD Detection

The amount of CPD was determined by ELISA and immunofluorescence microscopy, as described previously [42]. For ELISA, genomic DNA was reacted with anti-CPD antibody (Cosmo Bio, Tokyo, Japan). For immunofluorescence microscopy, cells were seeded onto 4-well chamber slides at 0.35 × 10^5^ cells per cm^2^ (0.6 × 10^5^ cells/well). The cells were treated with horse oil (0.312%), and after 1 h exposure to UVB, the cells were incubated at 37 °C for an additional 1 h. After fixation, permeabilization, and denaturation of the DNA, the cells were blocked with FBS, exposed to anti-CPD antibody and Alexa Fluor 594-conjugated F(ab′)2 fragment of anti-mouse IgG, and transferred to microscope slides in DAPI containing mounting medium (Vector Laboratories, Burlingame, CA, USA). Images were collected on a Zeiss confocal microscope using the LSM 510 program.

### 4.12. Mitochondrial Membrane Potential (Δψm) Analysis

Cells were treated with mitochondrial membrane permeant dye JC-1 (10 μg/mL) for 15 min at 37 °C. Next, the cells were washed with PBS, mounted on a microscope slide, and analyzed under an FV1200 laser-scanning microscope [43].

### 4.13. DNA Fragmentation Detection

Cellular DNA fragmentation was assessed by detecting the degree of cytoplasmic histone-associated DNA fragmentation with a diagnostics kit (Roche, Basel, Switzerland) according to the manufacturer’s protocol.

### 4.14. Nuclear Staining with Hoechst 33342

The DNA-specific fluorescent dye Hoechst 33342 (1.5 µL, 10 mg/mL stock) was added to each well, and cells were incubated at 37 °C for 10 min. Stained cells were observed under a fluorescence microscope equipped with a CoolSNAP-Pro color digital camera (Media Cybernetics, Rockville, MD, USA). The extents of nuclear condensation and apoptotic body formation were evaluated. The apoptotic index was calculated as follows: (number of apoptotic cells in treated group/total number of cells in treated group)/(number of apoptotic cells in control group/total number of cells in control group).

### 4.15. Western Blot Analysis

Thirty micrograms of protein were electrophoresed on 12% sodium dodecyl sulfate-polyacrylamide gels and transferred onto nitrocellulose membranes. The membranes were incubated with primary antibodies, followed by horseradish peroxidase-conjugated secondary antibody conjugates. Protein bands were visualized by using a Western blotting detection kit. Primary antibodies against PARP, caspase-3, caspase-9, phospho-JNK1/2, phospho-p38, and phospho-ERK1/2 were purchased from Cell Signaling Technology (Danvers, MA, USA); primary antibodies against Bax, Bcl-2, MMP-1, and actin were purchased from Santa Cruz Biotechnology (Dallas, TX, USA); and primary antibodies against MMP-2 and MMP-9 were obtained from Abcam (Cambridge, UK).

### 4.16. UV/visible Light Absorption Analysis

The stock solutions of horse oil were diluted in dimethyl sulfoxide at a ratio of 1:1000 (*v*/*v*). The solutions were scanned with UV light (range 200–400 nm) using a Biochrom Libra S22 UV/Visible spectrophotometer (Biochrom, Cambridge, UK).

### 4.17. MMP-1 Activity Assay

MMP-1 activity was measured by using a Fluorokine^®^ E human active MMP-1 assay kit (R&D Systems, Minneapolis, MN, USA) according to the manufacturer’s protocol.

### 4.18. Chromatin Immunoprecipitation (ChIP) Assay

ChIP assays were performed using the SimpleChIP™ enzymatic chromatin IP kit (Cell Signaling Technology) as previously described [44]. Immuno-precipitated DNA fragments were purified on spin columns and the recovered DNA was subjected to 35 cycles of PCR. Specific primers targeting the MMP-1 gene promoter (−67 to +94 from the transcription start site of the MMP-1 gene, Bionics) were as follows: sense 5′-CCTCTTGCTGCTCCAATATC-3′ and antisense 5′-TCTGCTAGGAGTCACCATTTC-3′. The PCR products were separated on an agarose gel, DNA bands were stained with EtBr, and the results were analyzed with ImageJ software (NIH, Bethesda, MD, USA).

### 4.19. Data Analysis

Data are expressed as the mean ± standard error (SE). Means were compared by analysis of variance followed by Tukey’s post-hoc test. A *p*-value < 0.05 was considered significant.

## 5. Conclusions

Our results show that the UVB protective effects of HO-I and HO-II were slightly but not significantly different. In summary, horse oil prevents UVB-stimulated oxidative stress by absorbing UVB radiation and removing ROS, reducing cell damage, and preventing cell death and aging. Therefore, the present data support that horse oil is a promising skin protectant for preventing and treating skin diseases associated with oxidative stress.

## Figures and Tables

**Table 1:** Fatty acid contents of horse oils.

Fatty Acid	HO-I (%)	HO-II (%)
C10:0 Capric acid		0.1
C12:0 Lauric acid	0.2	2.1
C14:0 Myristic acid	3.5	5.7
C16:0 Palmitic acid	23.8	29.4
C18:0 Stearic acid	2.8	4.8
Saturated fatty acid	30.3	42.1
C14:1 Myristoleic acid	0.4	0.3
C16:1 Palmitoleic acid	7.9	5.2
C18:1 Oleic acid	37.7	32.5
C18:2 Linoleic acid	19.7	15.0
C18:3 Linolenic acid	2.5	3.1
C20:1 Gadoleic acid	0.7	0.7
C20:2 Eicosadienoic acid	0.4	0.3
Unsaturated fatty acid	69.3	57.1
Unknown	0.4	0.8
Total	100	100

## Abbreviations

AP-1	Activation protein 1
CPD	Cyclobutane pyrimidine dimer
DPPH	2,2-Diphenyl-1-picrylhydrazyl
DPPP	Diphenyl-1-pyrenylphosphine
ERK1/2	Extracellular signal-regulated kinase 1/2
H_2_DCFDA	2′,7′-dichlorodihydrofluorescein diacetate
H_2_O_2_	Hydrogen peroxide
JNK1/2	c-Jun N-terminal kinase 1/2
MMP	Matrix metalloproteinase
MTT	Thiazolyl blue tetrazolium bromide
NAC	*N*-acetylcysteine
ROS	Reactive oxygen species
UV	Ultraviolet
